# A standardized fecal microbiota transplantation protocol enables consistent, microbiota-driven colitis in IL-10-deficient mice

**DOI:** 10.3389/fmicb.2026.1842264

**Published:** 2026-06-24

**Authors:** Prabhdeep Kaur, Jesús Rivera-Nieves

**Affiliations:** 1 Division of Gastroenterology, Department of Medicine, University of California San Diego, CA, United States; 2 Gastroenterology Section, San Diego VA Medical Center, San Diego, CA, United States

**Keywords:** colitis model, fecal microbiota transplantation, IL-10-deficient mice, inflammatory bowel disease, microbiota

## Abstract

Gut microbiota dysbiosis is a central feature of inflammatory bowel disease (IBD), yet experimental systems that enable controlled investigation of microbiota-driven inflammation remain limited. In interleukin-10-deficient (*Il*10^−/−^) mice, intestinal inflammation is strictly dependent on the presence of commensal microbiota; however, disease onset and severity are highly variable, reflecting differences in microbial composition across environments. To overcome this limitation, pharmacologic approaches such as piroxicam administration have been widely used to synchronize disease, but these methods introduce epithelial injury and non-microbiota-dependent inflammatory pathways that confound mechanistic interpretation. Here, we describe a standardized fecal microbiota transplantation (FMT) protocol that enables controlled microbiota-driven induction of colitis in *Il*10^−/−^ recipient mice without the use of chemical triggers. In this model, recipient mice aged 8–10 weeks receive fecal microbiota via oral gavage from either colitic *Il*10^−/−^; *Itgb*7^−/−^ double knockout (DKO) donor mice or non-colitic young *Il*10^−/−^ controls. The DKO donors exhibit impaired mucosal immune regulation and reduced IgA responses, features associated with the emergence of a colitogenic microbial community. Repeated FMT administration over 9 weeks promotes uniform disease induction and reduces variability in disease kinetics across experimental cohorts. Importantly, this approach preserves microbiota-driven disease mechanisms while improving experimental consistency compared with conventional spontaneous *Il*10^−/−^ models and avoids the confounding effects of pharmacologic synchronization. The protocol is compatible with downstream histological, immunological, and microbiome analyses and provides a practical platform for investigating host-microbiota interactions and microbiome-targeted therapeutic strategies in IBD.

## Graphical Abstract

**Figure:**
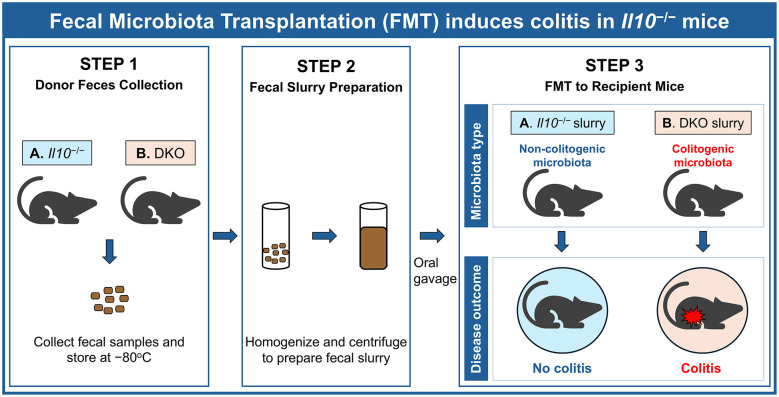
Fecal Microbiota Transplantation (FMT) induces colitis in Il10−/− mice. The FMT experiment consists of three main steps. (1) Donor feces collection: Fecal pellets were collected from young Il10−/− mice (non-colitic donors) or colitic Il10−/−; Itgb7−/− double knockout (DKO) donor mice and stored at −80 °C until processing. (2) Fecal slurry preparation: Frozen fecal pellets were thawed on ice, homogenized in phosphate-buffered saline (PBS), centrifuged, and resuspended to generate a fecal slurry. (3) FMT administration: Il10−/− recipient mice received 200 μL fecal slurry by oral gavage three times per week for 9 weeks. Transfer of microbiota from colitic DKO donors induced colitis in Il10−/− recipients, whereas microbiota from young Il10−/− donors did not induce colitis. This protocol enables controlled transfer of colitogenic microbiota and provides a microbiota-driven method to induce colitis in genetically susceptible hosts.

## 1 Introduction

Inflammatory bowel disease (IBD), encompassing ulcerative colitis (UC) and Crohn's disease (CD), comprises chronic inflammatory disorders of the gastrointestinal tract driven by complex interactions among host genetics, environmental factors, and the intestinal microbiota. A central feature of IBD pathogenesis is dysbiosis of the gut microbial community and the loss of immune tolerance to commensal organisms (Sartor, 2008). Experimental models have been instrumental in defining these relationships, demonstrating that intestinal inflammation can arise from aberrant host responses to otherwise non-pathogenic microbiota.

Seminal work established that interleukin-10-deficient (*Il*10^−/−^) mice develop spontaneous colitis that is strictly dependent on the presence of commensal bacteria (Kühn et al., 1993). Germ-free *Il*10^−/−^ mice remain disease-free, whereas colonization restores susceptibility to intestinal inflammation (Sellon et al., 1998; Berg et al., 2002). These studies provided direct evidence that the microbiota is necessary for disease initiation in a genetically susceptible host. Subsequent studies further demonstrated that disease severity is influenced by microbial composition rather than the presence of a single pathogen, supporting the concept that colitogenicity reflects properties of the microbial community as a whole (Sartor, 2008).

Despite these advances, a major limitation of the *Il*10^−/−^ model is the variable and asynchronous development of colitis under conventional housing conditions (Sellon et al., 1998; Berg et al., 1996; Kullberg et al., 1998). Disease penetrance, onset, and severity differ substantially across facilities and experimental cohorts, largely reflecting differences in microbial exposure and community structure. Disease severity is also influenced by genetic background, with BALB/c mice typically exhibiting more severe inflammation than C57BL/6 mice (Sartor, 2008; Berg et al., 2002; Ikenoue et al., 2005). This variability has limited the consistency of the model and constrained its utility for mechanistic studies. More broadly, many experimental colitis models introduce confounding mechanisms that complicate interpretation of host-microbiota interactions (Strober et al., 2002). In particular, models that rely on chemical injury or pharmacologic triggers often combine epithelial damage, immune activation, and microbial responses, making it difficult to isolate microbiota-specific contributions to disease pathogenesis.

To overcome the variability of the *Il*10^−/−^ model, pharmacologic approaches such as administration of the non-steroidal anti-inflammatory drug (NSAID) piroxicam have been widely adopted to synchronize disease onset. Initial studies demonstrated that NSAID treatment induces rapid and highly penetrant colitis in *Il*10^−/−^ mice (Berg et al., 2002). However, subsequent work has shown that piroxicam accelerates disease through mechanisms that include epithelial barrier disruption, increased epithelial apoptosis, and enhanced bacterial translocation across the mucosa (Berg et al., 2002; Holgersen et al., 2014). These findings indicate that the piroxicam-accelerated model reflects a combination of microbiota-dependent inflammation and chemically induced barrier injury. While effective in synchronizing disease, this approach introduces non-microbiota-dependent pathways that confound interpretation of microbiota-specific effects. Together, these limitations highlight the need for experimental systems that preserve microbiota-driven disease mechanisms while improving experimental consistency.

The composition of the donor microbiota is a key determinant of disease induction in transfer-based models. In prior work, we characterized interleukin-10 and integrin β7 double-knockout (*Il*10^−/−^; *Itgb*7^−/−^; hereafter referred to as double knockout [DKO] mice) as a model of severe intestinal inflammation associated with impaired mucosal immune regulation and defective lymphocyte trafficking (Tyler et al., 2022). These mice exhibit markedly reduced IgA responses, reflecting the combined effects of IL-10 deficiency on immunoglobulin class switching and β7 integrin deficiency on intestinal immune cell homing. The resulting disruption of mucosal immune homeostasis is associated with the emergence of a highly colitogenic microbial community. Notably, fecal material derived from these mice induces colitis when transferred into *Il*10^−/−^ recipients, suggesting that this microbial community possesses enhanced inflammatory potential. However, the precise microbial or functional features that confer this heightened colitogenicity remain incompletely defined.

Here, we describe a standardized fecal microbiota transplantation (FMT) protocol that enables controlled transfer of defined microbial communities into *Il*10^−/−^ recipient mice. By using microbiota derived from DKO donors, this approach provides a microbiota-driven method to induce reproducible colitis while avoiding the confounding effects of pharmacologic synchronization. This model improves consistency of disease induction while preserving microbiota causality and provides a robust platform for investigating host-microbiota interactions and microbiome-targeted interventions in IBD.

## 2 Materials and equipment

All reagents, mouse strains, and equipment used are listed in Table 1.

**Table 1:** Key resources.

Resource	Source	Identifier
*Il*10^−/−^ mice (B6.129P2-Il10/J)	The Jackson Laboratory, Bar Harbor, ME, USA	002251
Integrin β7^−/−^ mice (C57BL/6-Itgb7tm1Cgn/J)	The Jackson Laboratory, Bar Harbor, ME, USA	002965
*Il*10^−/−^; *Itgb*7^−/−^ mice (Double knockout)	Generated in-house	—
Phosphate-buffered saline (PBS)	Gibco, Thermo Fisher Scientific, Waltham, MA, USA	A1286301
Glycerol	Thermo Fisher Scientific, Waltham, MA, USA	17904
Cell strainer (70 μm)	Thermo Fisher Scientific, Waltham, MA, USA	22-363-548
Gavage needle (20G, 1.5″)	Thermo Fisher Scientific, Waltham, MA, USA	01-208-87
Syringes (1 mL)	BD Biosciences, Franklin Lakes, NJ, USA	309659
Microcentrifuge and conical tubes (1.5, 15, 50 mL)	Thermo Fisher Scientific/Genesee Scientific	Various
Centrifuge (≥4,000 × g)	Beckman Coulter, Brea, CA, USA	A99465
−80 °C freezer	SANYO/Panasonic Biomedical	—
CO_2_ chamber	UC San Diego Animal Facility	—
Weighing scale	OHAUS Corporation, Parsippany, NJ, USA	—
Plate reader	BioTek Instruments (Agilent), Winooski, VT, USA	—

Reagents, mouse strains, and consumables used for the fecal microbiota transplantation (FMT) protocol. Vendor names and catalog numbers are provided to facilitate reproducibility.

## 3 Step-by-step protocol: fecal microbiota transplantation for inducing colitis

### 3.1 Mouse breeding and housing

Estimated time: Ongoing.

*Il*10^−/−^ and *Itgb*7^−/−^ mice on a C57BL/6 background were obtained from The Jackson Laboratory (stock numbers 002251 and 002965, respectively) and bred in-house. *Il*10^−/−^; *Itgb*7^−/−^ double knockout (DKO) mice were generated by intercrossing and breeding to homozygosity for both alleles. Genotypes were confirmed by PCR-based genotyping (Transnetyx Inc., Cordova, TN, USA). Donor mice (*Il*10^−/−^ and DKO) and recipient mice (*Il*10^−/−^) were maintained under specific pathogen-free (SPF) conditions in a controlled environment (20-23 °C; 12-h light/dark cycle) with *ad libitum* access to irradiated chow (Envigo Teklad Global 2920X; Envigo, Indianapolis, IN, USA) and autoclaved water. Bedding and enrichment materials were standardized across cages, and cage changes were performed weekly under a hood. Recipient mice were randomly assigned to experimental groups. Both male and female mice were used in experiments; sex was balanced across treatment groups when possible. No consistent sex-specific differences in disease severity were observed. Mice were housed at a density of 5 mice per cage. Donor and recipient mice were maintained on separate racks to minimize unintended microbial transfer prior to FMT.

*Notes*:

1. Breeding and genotyping should be performed routinely to generate age-matched cohorts.
2. To minimize microbiota-driven variability, mice should be cohoused by treatment group from weaning whenever possible.
3. Experimental and non-experimental animals should be housed separately to limit unintended microbial transfer.

### 3.2 Donor fecal sample collection and storage

Estimated time: 1–2 h.

Fecal pellets were collected from genotypically confirmed donor mice:

#### 3.2.1 Non-colitic donors

Non-colitic donor mice were defined as *Il*10^−/−^ mice younger than 12 weeks of age with no clinical evidence of intestinal inflammation. These mice exhibited normal stool consistency with well-formed pellets, stable body weight relative to age-matched controls, and normal activity and grooming behavior. Mice with loose stool, diarrhea, weight loss, rectal prolapse, reduced activity, or poor body condition were excluded from the non-colitic donor group.

#### 3.2.2 Colitic donors

Colitic donor mice were *Il*10^−/−^; *Itgb*7^−/−^ double knockout (DKO) mice older than 12 weeks of age with clinical evidence of colitis as reported earlier (Tyler et al., 2022). Donors were identified based on the presence of clinical signs consistent with intestinal inflammation, including loose stool or diarrhea, weight loss, rectal prolapse, or reduced body condition and activity. Only mice with confirmed *Il*10^−/−^; *Itgb*7^−/−^ genotype and clear clinical signs of disease were used as colitic donors, while mice without clinical evidence of colitis were excluded.

To ensure that donor mice had active disease, colitis was confirmed by histologic evaluation in a subset of donor mice from each cohort prior to fecal collection and use as microbiota donors. To reduce donor-to-donor variability, fecal pellets were collected from multiple mice meeting the inclusion criteria within the same donor group and pooled. Specifically, freshly voided fecal pellets were collected from 2–3 cages per donor group and combined into a single preparation using sterile forceps. To preserve obligate anaerobic bacteria, fecal material was processed rapidly on ice with minimized oxygen exposure using sealed tubes. All sample preparation was performed under standard laboratory conditions; consistent disease induction suggests preservation of functionally relevant microbial communities. For FMT administrations, aliquots from pooled donor material were prepared and stored to minimize freeze-thaw cycles and maintain consistency across time points. Samples were snap-frozen on dry ice and stored at −80 °C.

*Notes*:

1. Collect only freshly voided pellets from the same cages and pool them; avoid collection from bedding.
2. Use aseptic technique throughout sample collection.
3. Aliquot samples prior to freezing to prevent repeated freeze-thaw cycles.
4. Record donor cage, genotype, age, and collection date for traceability.
5. Optional: For long-term storage, fecal material may be suspended in phosphate-buffered saline (PBS) containing 20% glycerol prior to freezing.

### 3.3 Preparation of donor fecal slurry

Estimated time: 30–45 min.

Fecal pellets were thawed on ice and homogenized in sterile, cold PBS at a ratio of approximately 100 mg feces per 1 mL PBS using vortexing or mechanical disruption. Homogenates were centrifuged at 4,000 × *g* for 15 min at 4 °C. The supernatant was removed, and the pellet was resuspended in sterile PBS to concentrate the microbial fraction. The resulting suspension was adjusted to a final volume of 200 μL per original fecal pellet and passed through a 70 μm cell strainer to obtain a uniform slurry. To standardize inoculum density across preparations, optical density at 600 nm (OD_600_) was measured. Preparations with OD_600_ values between 1.5 and 2.0 were used for FMT administration.

*Notes*:

1. Maintain samples on ice throughout preparation. For fecal pellets previously suspended in PBS containing 20% glycerol and stored at −80 °C, thaw samples on ice and centrifuge at 4,000 × *g* for 15 min at 4 °C. Discard the supernatant and resuspend the pellet in sterile PBS to a final volume of 200 μL per original fecal pellet. Pass the suspension through a 70 μm cell strainer to generate a uniform fecal slurry prior to gavage.
2. Minimize oxygen exposure during sample preparation to preserve obligate anaerobes.
3. Use sterile equipment and aseptic technique.
4. Thoroughly mix fecal slurry before each administration to ensure uniformity.
5. Record OD_600_ values and preparation details for quality control.

### 3.4 Fecal microbiota transplantation (FMT)

Estimated time: approximately 10 min per mouse.

Duration: 9 weeks (Three FMT administrations per week).

**Recipient mice:** Recipient mice were *Il*10^−/−^ mice 8–10 weeks of age with confirmed genotype and no clinical evidence of intestinal inflammation at baseline. Only mice with normal stool consistency, stable body weight, normal activity, and no rectal prolapse were included. Mice with loose stool, weight loss, reduced activity, poor body condition, or other signs of pre-existing colitis were excluded prior to FMT. Mice were maintained on standard diet. Both male and female mice were used and were randomly assigned to the following experimental groups prior to FMT.

- **Control group (non-colitis):** Received fecal slurry from non-colitic *Il*10^−/−^ donors.
- **Inflamed group (colitis):** Received fecal slurry from colitic DKO donors.

A total of 200 μL fecal slurry was administered via oral gavage using sterile 1 mL syringes and 20G gavage needles. FMT was performed three times per week for 9 consecutive weeks.

*Notes*:

1. Use separate gavage syringes, needles and fresh gloves for each group to prevent cross-contamination.
2. Gently restrain mice in an upright position and insert the gavage needle carefully to avoid injury.
3. Perform all administrations at consistent times of day to minimize circadian variation.
4. Remove fecal slurry from ice approximately 20 min before administration and mix between mice to prevent sedimentation.
5. Record mouse ID, dosing time, and any procedural complications.

### 3.5 Monitoring and sample collection

Timing: Weekly; endpoint at 9 weeks or upon reaching humane endpoints.

#### 3.5.1 Clinical monitoring

Mice were monitored daily for signs of disease, including weight loss, reduced activity, piloerection, and changes in stool consistency. Body weight was recorded weekly. Mice were euthanized upon reaching predefined humane endpoints, including ≥20% loss of initial body weight or severe clinical signs.

#### 3.5.2 Sample collection

- Fecal samples: Collected weekly as freshly voided pellets and stored at −80 °C for downstream analyses.
- Blood: Collected via submandibular vein puncture and centrifuged at 2,000 × *g* for 10 minutes to isolate serum, which was stored at −80 °C.

*Notes*:

1. Standardize sample collection timing across all mice.
2. Use sterile tools to prevent contamination.
3. Aliquot samples to minimize freeze-thaw cycles.
4. Optional: A disease activity index (DAI), incorporating stool consistency and general condition, may be recorded for mice.

### 3.6 Euthanasia and sample collection

Estimated time: 10–20 min per mouse.

Mice were euthanized upon reaching humane endpoints (≥20% weight loss or severe clinical signs) or at study completion. Euthanasia was performed by CO_2_ inhalation followed by cervical dislocation as a secondary method. Following disinfection with 70% ethanol, samples can be collected via ventral midline incision for the following downstream analyses:

- Histopathology: Colon (primary tissue), cecum (optional).
- Flow cytometry: Peyer's patches, mesenteric lymph nodes, cecal patch.
- Gene expression: Colon epithelial scrapings (stored at −80 °C).
- ELISA: Serum and fecal samples.
- Microbiome analysis: Fecal samples.

*Notes*:

1. Use sterile instruments and disinfect between mice.
2. Process tissues immediately to preserve integrity.

## 4 Expected outcomes

FMT from colitic DKO donors results in consistent induction of colitis in *Il*10^−/−^ recipient mice. In contrast to conventional *Il*10^−/−^ models, in which disease onset is variable and often asynchronous, this protocol promotes uniform disease onset and reduces variability in disease kinetics across experimental cohorts.

Clinical signs typically emerge within 2–4 weeks following initiation of FMT and include progressive weight loss, loose or unformed stools, reduced activity, and, in some cases, rectal prolapse. Disease progression can be monitored longitudinally using body weight and clinical scoring systems, including disease activity indices. Histologically, colonic tissue demonstrates characteristic features of inflammatory colitis, including epithelial hyperplasia, crypt architectural distortion, mucosal ulceration, and infiltration of inflammatory cells (Figure 1). These features can be quantified using established histopathological scoring systems (Dieleman et al., 1998). At the immunological level, increased mucosal immune activation is typically observed, including changes in immunoglobulin A (IgA) levels in stool and serum, as well as expansion of immune cell populations within intestinal and lymphoid tissues. These phenotypic and immunological changes are quantitatively reflected by differences in body weight, histological inflammation scores, and fecal and serum IgA levels between experimental groups (Figure 2). The magnitude of these responses may vary depending on donor microbiota composition and host factors. Notably, variability in disease severity may still occur due to differences in donor microbiota composition, host factors, and housing conditions; however, this variability is generally reduced relative to conventional *Il*10^−/−^ models. In our experience, this protocol results in reproducible induction of colitis in nearly 100% of recipient mice, as defined by histological inflammation and associated clinical and immunological parameters.

**Figure 1:**
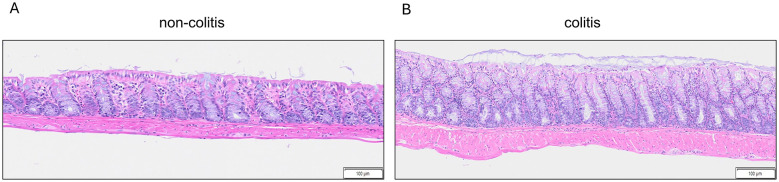
Fecal microbiota transplantation (FMT) from colitic donors promotes intestinal inflammation in *Il*10^−/−^ recipient mice. Representative hematoxylin and eosin (H&E)-stained colon sections are shown from *Il*10^−/−^ recipient mice that received fecal microbiota from non-colitic *Il*10^−/−^ donors **(A)** or colitic DKO donors **(B)**. Mice receiving colitic donor microbiota exhibit features of inflammatory colitis, including epithelial hyperplasia, crypt architectural distortion, and inflammatory cell infiltration. Scale bar, 100 μm. DKO, *Il*10^−/−^; *Itgb*7^−/−^ double knockout mice.

**Figure 2:**
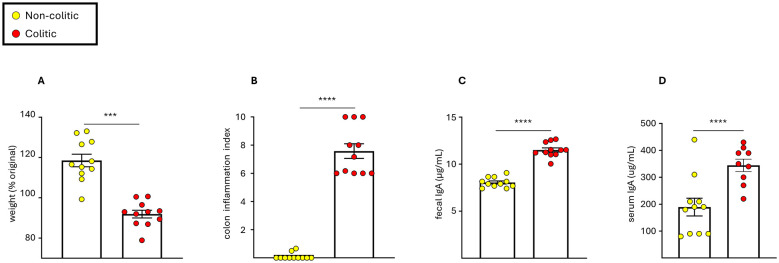
Quantitative validation of FMT-induced colitis in *Il*10^−/−^ recipient mice. **(A)** Body weight expressed as percentage of initial weight in recipient mice following fecal microbiota transplantation from non-colitic *Il*10^−/−^ donors (yellow) and colitic *Il*10^−/−^; *Itgb*7^−/−^ (DKO; red) donors. **(B)** Histological inflammation scores of the colon, demonstrating increased inflammation in mice receiving colitic donor microbiota. **(C)** Fecal IgA levels. **(D)** Serum IgA levels. Data are presented as mean ± SEM (n ≥ 9 per group). Statistical significance was determined using unpaired two-tailed Student's *t*-test (with Welch's correction). Significance is indicated as ***P < 0.001; ****P < 0.0001. FMT, fecal microbiota transplantation; DKO, double knockout.

Collectively, these findings demonstrate that transfer of colitogenic microbiota provides a controlled, microbiota-driven approach to induce colitis in genetically susceptible hosts while maintaining experimental control over disease initiation and progression.

## 5 Limitations

1. Donor variability: Microbial composition differences can influence disease severity. Even with pooling strategies, variability in donor microbiota remains an inherent feature of FMT-based models.
2. Housing effects: Cage-specific microbiota may affect outcomes. Strict control of housing conditions is required to minimize inter-cage variability. Environmental factors, including cage effects and facility-specific microbiota, may influence disease penetrance and should be carefully controlled.
3. Model specificity: *Il*10^−/−^ mice reflect immune dysregulation and microbiota-dependent inflammation but may not capture all aspects of human IBD. Additionally, the reliance on *Il*10^−/−^; *Itgb*7^−/−^ donor mice may limit accessibility for some laboratories, although similar approaches could be adapted using alternative donor microbiota.
4. Facility dependence: Microbiota composition may vary across institutions, potentially affecting disease penetrance and reproducibility across independent facilities.
5. Temporal limitation: The model primarily reflects subacute to chronic colitis over a defined experimental window.
6. Microbiota characterization: Microbial engraftment was inferred from disease transfer rather than directly confirmed by microbiome sequencing, which represents a limitation of the current study.

## 6 Troubleshooting

Successful execution of this protocol requires strict attention to donor selection, sample preparation, administration techniques, and housing conditions. Variability in any of these steps can affect microbial engraftment and colitis severity. Table 2 summarizes common technical and biological issues, along with practical solutions to improve reproducibility and animal welfare.

**Table 2:** Troubleshooting guide for fecal microbiota transplantation (FMT)-induced colitis protocol in *Il*10^−/−^ mice.

Step	Problem	Possible cause	Suggested solution
1. Mouse housing and breeding	Variable disease onset or severity	Cage effects or mixed housing	Cohouse by treatment group and avoid mixing groups
	Low breeding efficiency	Poor pairing or error in genotyping	Perform regular breeding checks and ensure timely genotyping
2. Donor fecal collection and storage	Poor colonization or colitogenic effect	Sample degradation (freeze-thaw)	Snap-freeze, store samples at −80 °C and make aliquots
	Sample contamination	Bedding contamination	Collect freshly voided fecal pellets with sterile tools and store immediately
3. Fecal slurry preparation	Low OD_600_ (< 1.5)	Insufficient fecal sample or dilution errors	Increase pellet: PBS ratio and homogenize thoroughly
	High OD_600_ (>2.0) or visible clumps	Incomplete homogenization	Adjust PBS: pellet ratio and homogenize thoroughly
4. FMT gavage	Gavage injury	Improper gavage technique	Oral gavage by trained laboratory personnel only
	Inconsistent dosing	Sedimentation of slurry	Vortex/pipette mix fecal slurry between mice to maintain uniform distribution
	Variable disease induction	Donor variability or timing differences	Use same donor batch and administer FMT at the same time of day
5. Monitoring and sample collection	Poor sample quality	Contamination or degradation during handling	Collect samples with sterile tools and store immediately
	Premature mortality	Severe inflammation or gavage complications	Monitor mice regularly, apply humane endpoints, adjust FMT frequency and check gavage technique
	Incomplete blood or tissue collection	Inexperienced personnel or tissue damage during collection	Sample collection by trained personnel only and process samples immediately
6. Outcomes	No colitis phenotype	Poor donor sample quality or ineffective FMT	Confirm donor disease phenotype, verify OD_600_ and repeat with fresh batch
	Variable histological findings	Inconsistent tissue handling	Sample collection by trained personnel and standardize sample processing methods

Common technical and biological issues encountered during the protocol are summarized with potential causes and recommended solutions to improve reproducibility and experimental consistency.

## 7 Discussion

Here, we describe a standardized FMT protocol that enables controlled and consistent induction of colitis in *Il*10^−/−^ recipient mice through transfer of defined microbial communities. This approach addresses a central limitation of the *Il*10^−/−^ model, the variable and asynchronous development of disease under conventional housing conditions, while preserving the microbiota-dependent nature of intestinal inflammation (Sellon et al., 1998; Berg et al., 2002).

A major challenge in studying microbiota-driven disease has been the trade-off between biological relevance and experimental consistency. While *Il*10^−/−^ mice provide a physiologically relevant model in which inflammation is dependent on commensal microbiota, disease onset and severity are highly influenced by environmental microbial exposure and community composition (Sartor, 2008). This variability limits reproducibility and complicates mechanistic studies of host-microbiota interactions. Compared with germ-free or spontaneous *Il*10^−/−^ models, the present approach enables controlled and reproducible disease induction without requiring specialized housing or pharmacologic triggers, thereby improving experimental accessibility and consistency.

To overcome this limitation, pharmacologic approaches such as piroxicam administration have been widely adopted to synchronize disease onset (Berg et al., 2002; Holgersen et al., 2014). However, these models introduce epithelial injury and non-microbiota-dependent inflammatory pathways, including disruption of barrier integrity and enhanced bacterial translocation, thereby complicating interpretation of microbiota-specific effects (Holgersen et al., 2014). More broadly, many experimental colitis models combine epithelial injury, immune activation, and microbial responses, making it difficult to specifically examine the role of microbiota-driven mechanisms (Table 3) (Strober et al., 2002).

**Table 3:** Comparison of commonly used murine colitis models and microbiota-driven approaches.

Model	Primary trigger	Microbiota dependence	Reproducibility	Key limitations
DSS colitis (Wirtz et al., 2007)	Chemical epithelial injury	Indirect	High	Acute injury, limited microbiota specificity
TNBS colitis (Wirtz et al., 2007; Morris et al., 1989)	Hapten-mediated immune response	Indirect	Moderate	Variable severity, chemical toxicity
PAC *Il*10^−/−^ (Holgersen et al., 2014)	NSAID-accelerated inflammation	Limited	High	Exogenous inflammatory trigger
Germ-free *Il*10^−/−^ colonization (Sellon et al., 1998)	Defined microbiota	High	Variable	Specialized facilities required
FMT-based *Il*10^−/−^ model (this protocol)	Donor microbiota	High	High	Donor variability, housing effects

Experimental colitis models are compared based on their primary trigger, degree of microbiota dependence, reproducibility, and key limitations. DSS, dextran sulfate sodium; TNBS, 2,4,6-trinitrobenzene sulfonic acid; PAC, piroxicam-accelerated colitis; *Il*10^−/−^, interleukin-10-deficient; NSAID, non-steroidal anti-inflammatory drug; FMT, fecal microbiota transplantation.

The present protocol provides a microbiota-driven alternative to pharmacologic synchronization. By transferring fecal microbiota derived from colitic donors, this approach enables uniform disease induction while maintaining the central role of microbial communities in driving inflammation. In this protocol, histologic inflammation serves as the primary endpoint to confirm successful disease induction. However, additional analyses, including flow cytometry, immunoglobulin measurements, gene expression analysis, and microbiome sequencing, can be incorporated depending on the experimental objectives. In this context, FMT can be viewed as a biologic approach to standardize microbial exposure and reduce variability in disease kinetics in *Il*10^−/−^ mice.

An important feature of this model is the use of *Il*10^−/−^; *Itgb*7^−/−^ double knockout mice as donors. These mice exhibit impaired mucosal immune regulation and defective lymphocyte trafficking, resulting in markedly reduced IgA responses and disruption of intestinal immune homeostasis (Tyler et al., 2022). IgA plays a critical role in immune exclusion and containment of commensal bacteria at the mucosal surface, and its deficiency may permit expansion of microbial communities with enhanced inflammatory potential (Sartor, 2008). Consistent with this concept, fecal material from these donors induces colitis when transferred into non-colitic *Il*10^−/−^ recipients. However, the precise microbial or functional features responsible for this increased colitogenicity remain incompletely defined and represent an important area for future investigation. While direct microbiota profiling was not performed in this study, prior work has demonstrated that microbiota derived from *Il*10^−/−^; *Itgb*7^−/−^ donors exhibits altered composition and colitogenic potential (Tyler et al., 2022). The consistent transfer of disease phenotype across experiments further supports successful microbial engraftment.

FMT has also been translated into clinical practice, where it is now widely used for the treatment of recurrent *Clostridium difficile* infection, demonstrating high efficacy in restoring microbial balance and resolving disease (van Nood et al., 2013; Cammarota et al., 2017). In contrast, the therapeutic application of FMT in inflammatory bowel disease, particularly ulcerative colitis, has yielded variable results across clinical studies, with differences in donor selection, treatment protocols, and host factors contributing to heterogeneous outcomes (Paramsothy et al., 2017; Costello et al., 2019). These observations highlight both the central role of the microbiota in modulating intestinal inflammation and the challenges associated with controlling microbial composition in clinical settings. In this context, experimental systems that enable defined and controlled transfer of microbial communities are essential for dissecting the mechanisms underlying microbiota-driven disease and therapeutic response. Compared with chemically induced models such as dextran sulfate sodium (DSS) or 2,4,6-trinitrobenzene sulfonic acid (TNBS), and pharmacologically accelerated models such as piroxicam-treated *Il*10^−/−^ mice, this approach maintains a primary role for microbial community structure in disease initiation while improving experimental control (Strober et al., 2002; Holgersen et al., 2014). Standardization of donor material, pooling strategies, and repeated FMT administration further contribute to improved consistency across experimental cohorts. DSS, TNBS, and piroxicam-treated *Il*10^−/−^ mouse models introduce epithelial injury or exogenous inflammatory stimuli that may confound interpretation of microbiota-specific effects. In contrast, the FMT-based *Il*10^−/−^ model described here enables controlled transfer of donor microbial communities, providing a microbiota-driven approach to disease induction while improving experimental consistency relative to conventional spontaneous *Il*10^−/−^ models.

Several limitations should be considered. As with all microbiota transfer approaches, donor-to-donor variability in microbial composition can influence disease severity (Sartor, 2008). Housing conditions and inter-cage microbial transfer may also impact experimental outcomes, necessitating strict control of animal housing. In addition, while the *Il*10^−/−^ model captures key aspects of microbiota-driven inflammation, it does not fully recapitulate the heterogeneity of human IBD (Strober et al., 2002). Finally, the microbial taxa or functional pathways responsible for disease transfer remain to be defined. Despite these limitations, this protocol provides a practical and consistent framework for studying microbiota-driven intestinal inflammation.

In summary, this FMT-based *Il*10^−/−^ colitis model provides a controlled and microbiota-driven platform for inducing colitis in genetically susceptible hosts. By enabling standardized transfer of colitogenic microbial communities, this approach facilitates mechanistic studies of host-microbiota interactions and supports the development of microbiome-targeted therapeutic strategies in IBD.

## Data Availability

The raw data supporting the conclusions of this article will be made available by the authors, without undue reservation.
